# Single-cell dissection and multi-cohort validation identify a hypoxia-related prognostic signature with experimental verification in lung adenocarcinoma

**DOI:** 10.3389/fphar.2026.1845179

**Published:** 2026-06-24

**Authors:** Fan Zhang, Junyan Wang, Yu Xu, Minting Ma, Suju Wei, Chengyuan Liu

**Affiliations:** 1 Medical Oncology Department, The Fourth Hospital of Hebei Medical University, Shijiazhuang, Hebei, China; 2 General Practice Department, The Fourth Hospital of Hebei Medical University, Shijiazhuang, Hebei, China

**Keywords:** hypoxia, lung adenocarcinoma, prognostic model, single-cell RNA sequencing, tumor microenvironment, WGCNA

## Abstract

**Background:**

Lung adenocarcinoma (LUAD) shows marked molecular heterogeneity and diverse clinical outcomes. Tumor hypoxia is a key microenvironmental factor that promotes metabolic reprogramming, invasive phenotypes, therapeutic resistance, and immune remodeling. However, the cellular distribution of hypoxia signaling in LUAD and its value for robust prognostic stratification remain incompletely understood.

**Methods:**

We integrated single-cell RNA sequencing data, multi-cohort bulk transcriptomic data, and clinical follow-up information to investigate hypoxia-related features in LUAD. At the single-cell level, cells from a Gene Expression Omnibus LUAD cohort were annotated and hypoxia scores were calculated using ssGSEA. At the bulk level, weighted gene co-expression network analysis and differential expression analysis were combined to identify hypoxia-related key genes. Multiple algorithms were compared to construct a prognostic model, which was further validated in external cohorts. Clinical associations, pathway enrichment, immune infiltration, mutational features, and drug sensitivity were also analyzed. In addition, the top contributing gene was functionally validated *in vitro* through overexpression, proliferation, and wound-healing assays.

**Results:**

Single-cell analysis identified nine major cell populations and revealed marked heterogeneity in hypoxia signaling across cell types. Hypoxia scores were highest in macrophages and fibroblasts, intermediate in epithelial and endothelial cells, and generally low in lymphocyte-related populations. In bulk analyses, 103 hypoxia-related key genes were identified. A prognostic model derived from these genes effectively stratified patients into high-risk and low-risk groups in the TCGA training cohort and two independent external validation cohorts. High-risk patients had significantly poorer survival and more advanced clinical features. Functional analyses showed that the high-risk group was enriched for proliferation- and invasion-related programs, whereas the low-risk group showed enrichment in metabolism- and homeostasis-related pathways. Immune, mutational, and drug sensitivity analyses further supported distinct biological characteristics between risk groups. Moreover, overexpression of VWA1, the key gene with the highest contribution to the model, significantly promoted A549 cell proliferation and migration.

**Conclusion:**

This study systematically characterized the cellular heterogeneity of hypoxia signaling in LUAD and established a cross-cohort validated hypoxia-related prognostic model. The hypoxia-associated high-risk state was closely linked to poor prognosis and distinct functional and microenvironmental features. These findings provide new evidence for prognostic assessment and individualized therapeutic exploration in LUAD.

## 1 Introduction

Lung adenocarcinoma (LUAD) is one of the most common subtypes of non-small cell lung cancer (Wang et al., 2022). Although targeted therapy and immunotherapy have substantially improved the prognosis of a subset of patients, LUAD overall still exhibits marked molecular heterogeneity and pronounced variability in clinical outcomes (Jamal-Hanjani et al., 2017; Cancer Genome Atlas Research Network, 2014). Even among patients with the same clinical stage or similar driver genomic backgrounds, survival time and therapeutic responses can differ dramatically (Skoulidis and Heymach, 2019). This indicates that reliance on conventional staging systems or single molecular events is insufficient to capture the biological complexity of LUAD, thereby constraining further optimization of risk stratification and individualized treatment strategies (Vargas and Harris, 2016).

Tumor hypoxia is a key microenvironmental factor that drives LUAD progression and therapeutic resistance (Chen et al., 2023; Lee et al., 2020). Beyond reflecting a mismatch between tumor perfusion and metabolic demand, hypoxia can reshape energy metabolism, promote cell-cycle progression and stress adaptation, and induce state transitions such as epithelial–mesenchymal transition (EMT), thereby influencing the immune microenvironment and drug sensitivity at multiple levels (Petrova et al., 2018; Imai et al., 2003; Hubbi and Semenza, 2015; Barsoum et al., 2011). Hypoxia-associated transcriptional programs are frequently linked to aggressive phenotypes, poor prognosis, and resistance to radiotherapy and chemotherapy (Wang et al., 2021; Buffa et al., 2010). However, hypoxia signaling is not uniformly distributed within tumor tissues (Vaupel and Mayer, 2007). It may vary substantially across cell types and can be further amplified through the involvement of myeloid and stromal cells (Gilkes et al., 2014). Consequently, hypoxia scores derived solely from bulk transcriptomic data are susceptible to confounding by differences in cellular composition, and they cannot readily address key questions such as which cellular populations predominantly bear hypoxic programs and how hypoxia-related risk states are coupled with the tumor ecological niche (Iotchkova et al., 2019; Newman et al., 2015).

In recent years, single-cell transcriptomic technologies have provided a higher-resolution framework for dissecting cellular composition and functional states within the tumor microenvironment (Wu et al., 2021; Lambrechts et al., 2018). At the cellular level, these approaches enable characterization of the distribution of hypoxia-related transcriptional activity across distinct cell populations and facilitate the identification of potential state differences and coordinated interactions among malignant epithelial cells, immune cells, and stromal cells (Puram et al., 2017; Qian et al., 2020; Kum et al., 2018). On the other hand, single-cell datasets often have limited sample sizes and are not well suited for building stable clinical prognostic models (Kuksin et al., 2021; Lähnemann et al., 2020). In contrast, multi-cohort bulk transcriptomic datasets typically offer more comprehensive follow-up information and greater statistical power, making them more suitable for developing prognostic models with clinical generalizability (Newman et al., 2019; Subramanian et al., 2005). Therefore, integrating single-cell–anchored biological insights with the modeling strengths of bulk cohorts to establish a hypoxia-related stratification system that is both mechanistically interpretable and robust across cohorts holds clear scientific and translational value.

In this study, we integrated single-cell and multi-cohort bulk transcriptomic data to systematically evaluate hypoxia heterogeneity within the LUAD tumor microenvironment and to develop a hypoxia-centered prognostic stratification framework. Unlike previous bulk-only hypoxia signatures, this study first characterized the cellular distribution of hypoxia-related transcriptional activity at single-cell resolution, thereby providing biological context for the heterogeneity of hypoxia-related signals in clinical tumor samples. Bulk transcriptomic cohorts were then used to identify hypoxia-associated candidate genes and construct a clinically applicable prognostic model with external validation in independent GEO cohorts. In addition, we performed functional validation of VWA1, the top-ranked model gene, in LUAD cells. Therefore, the incremental value of this study lies in combining cell-level hypoxia heterogeneity, cross-cohort prognostic modeling, multi-dimensional biological interpretation, and preliminary experimental validation within a single analytical framework. This design provides a more interpretable and testable basis for hypoxia-related risk stratification in LUAD.

## 2 Materials and methods

### 2.1 Data sources

This study integrated single-cell transcriptomic data and multi-cohort bulk transcriptomic datasets to characterize the cellular composition of the LUAD tumor microenvironment, delineate hypoxia heterogeneity, and develop a hypoxia-related prognostic stratification model. Single-cell RNA sequencing data were obtained from the Gene Expression Omnibus (GEO) database under accession number GSE189357. Raw 10x Genomics gene expression matrices from nine LUAD-related samples (TD1–TD9) were downloaded and processed individually before integration. The TCGA-LUAD cohort served as the bulk transcriptomic training set, and GSE31210 and GSE50081 were used as external validation cohorts. Included samples were required to have complete expression matrices and overall survival information for model training and independent validation.

### 2.2 Data preprocessing

Bulk transcriptomic data were uniformly preprocessed prior to analysis. Raw counts, TPM, or other expression measures were normalized, followed by log_2_ (x + 1) transformation to mitigate the influence of extreme values on downstream analyses. Only genes with stable expression within each dataset were retained for subsequent analyses. Clinical annotations were checked for completeness, and samples with missing key follow-up fields were excluded. Overall survival (OS) was defined as the time from initial diagnosis to death or last follow-up. Survival status was coded as death or censoring. To improve cross-cohort comparability, the intersection of genes shared across cohorts was used for modeling. After log_2_ (x + 1) transformation, batch effects among bulk transcriptomic cohorts were corrected using the ComBat function in the sva package, and expression standardization were performed when necessary.

### 2.3 Single-cell RNA sequencing analysis

Single-cell RNA sequencing data were analyzed in R using Seurat (Stuart et al., 2019). A Seurat object was constructed for each sample, and low-quality cells were filtered based on quality control metrics, including the number of detected genes, UMI counts, and the proportion of mitochondrial transcripts. Cells were retained if they had more than 100 and fewer than 7,500 detected genes, more than 1,000 UMI counts, and less than 15% mitochondrial transcripts. Data were then normalized, and the top 2,000 highly variable genes were selected. After sample integration and batch correction using Harmony, dimensionality reduction and unsupervised clustering were performed. Two-dimensional visualization was generated using t-SNE to display the cellular landscape. Cell-type annotation was manually assigned based on canonical marker gene expression patterns and was further verified by visualization. Major cell populations identified for downstream analyses included T/NK cells, B cells, plasma cells, epithelial cells, macrophage cells, mast cells, fibroblasts, endothelial cells, and myeloid cells.

The hypoxia-related gene set was derived from the Molecular Signatures Database (MSigDB) Hallmark Hypoxia gene set, which contains curated genes involved in cellular responses to hypoxia and hypoxia-associated transcriptional programs. This gene set was selected because it provides a standardized and biologically interpretable representation of hypoxia activity and has been widely used in pathway-level transcriptomic analyses. Single-cell hypoxia scores were computed using ssGSEA based on this gene set (Lei et al., 2021). Scores were mapped onto the t-SNE space and compared across cell types to evaluate cellular heterogeneity of hypoxia signaling in the LUAD tumor microenvironment.

### 2.4 Identification of hypoxia-related modules and key genes

In the TCGA-LUAD cohort, a sample-level hypoxia score was calculated using the same MSigDB Hallmark Hypoxia gene set and was used as a continuous trait for weighted gene co-expression network analysis (WGCNA) to identify co-expression modules significantly associated with hypoxia status (Langfelder and Horvath, 2008). To connect the single-cell and bulk analyses, WGCNA was performed within the candidate gene space defined by hypoxia-associated epithelial marker genes identified from the single-cell analysis. Samples were first hierarchically clustered to assess expression profile consistency and to exclude potential outliers. The soft-thresholding power was selected using the pickSoftThreshold function according to the scale-free topology criterion. An unsigned co-expression network was then constructed using blockwiseModules with topological overlap matrix-based module detection. Stable modules were identified using dynamic tree cutting with minModuleSize = 50, and similar modules were merged using mergeCutHeight = 0.15. Hypoxia-related core modules were selected based on correlations between module eigengenes and the hypoxia score. Within the core modules, the relationship between module membership and gene significance was evaluated to support the identification of key candidate genes. In parallel, TCGA-LUAD samples were stratified by hypoxia score and differential expression analysis was performed to obtain hypoxia-associated differentially expressed genes (DEGs). The intersection of genes from the hypoxia-related core modules and DEGs was defined as the set of hypoxia-related key candidate genes for subsequent prognostic modeling and functional annotation.

### 2.5 Construction and validation of the hypoxia-related prognostic model

The TCGA-LUAD cohort was used as the training set, and GSE31210 and GSE50081 served as external validation sets. Based on the expression matrix of 103 hypoxia-related key candidate genes and OS follow-up information, survival prediction models were developed in the TCGA-LUAD training cohort. To reduce algorithm-specific bias and avoid relying on a single modeling strategy, multiple survival modeling algorithms and their combinations were systematically compared, including random survival forests (RSF), stepwise Cox regression, LASSO regression, ridge regression, elastic net regression, CoxBoost, gradient boosting models, partial least squares Cox regression, survival support vector machines, and SuperPC. For the RSF-based models, ntree was set to 1,000, nodesize was set to 5, and the log-rank splitting rule was used. For penalized regression models, including LASSO regression, ridge regression, and elastic net regression, λ was selected by 10-fold cross-validation using lambda. min. Penalized regression models and boosting-based models were tuned using cross-validation procedures, and model performance was evaluated in both the training cohort and two independent external validation cohorts. The final model was selected according to its overall predictive performance and cross-cohort stability rather than its apparent performance in the training cohort alone. Model performance was assessed using time-dependent ROC curves and C-index values. Because performance estimates derived from the training cohort may be optimistic, the results from GSE31210 and GSE50081 were considered more informative for evaluating model generalizability.

Model robustness was assessed using two complementary diagnostics: optimism-corrected internal validation via 1,000 bootstrap resamples (Harrell’s method) to quantify and adjust for overfitting in the apparent C-index and 1/3/5-year time-dependent AUCs, and calibration assessment at 1, 3, and 5 years in both training and external cohorts, evaluated using calibration slopes (from Cox regression on the model’s linear predictor) and observed-to-expected (O/E) ratios comparing Kaplan–Meier-estimated event rates with mean predicted probabilities.

### 2.6 Clinical utility evaluation

In the TCGA-LUAD cohort, distributions of clinicopathological characteristics were compared between high- and low-risk groups, including TNM stage and overall clinical stage. The stratification ability of the risk score was further assessed within clinical subgroups such as stage and age. Stratified Kaplan–Meier survival analyses were performed to compare OS between high-risk and low-risk groups within each subgroup, thereby evaluating model robustness across clinical contexts.

Cox proportional hazards models were applied to assess the effects of the risk score and clinical variables on OS. Univariate Cox regression was first performed to screen variables significantly associated with survival. Candidate variables were then included in multivariable Cox regression to determine independent prognostic factors. A nomogram was constructed based on independent prognostic factors to estimate individualized 1-, 3-, and 5-year OS probabilities. Calibration curves were used to assess agreement between predicted and observed outcomes, and discrimination was evaluated using the concordance index (C-index) and related metrics. Decision curve analysis (DCA) was conducted to compare the clinical net benefit of the nomogram with single-variable models across threshold probabilities.

### 2.7 Functional pathway analyses based on risk stratification

To investigate functional differences between high- and low-risk groups, gene set enrichment analysis (GSEA) was performed using pathway collections such as Hallmark gene sets. Gene set variation analysis (GSVA) was used to compute sample-level pathway activity scores and to evaluate global shifts in pathway activity across risk groups. Cox regression was further applied to assess associations between key pathway activities and OS, enabling identification of prognostically relevant functional programs.

### 2.8 Immune microenvironment analysis

To evaluate the relationship between risk stratification and the immune microenvironment, immune-related pathway activities were compared at the sample level, and deconvolution approaches were applied to estimate immune cell infiltration. Differences in immune cell composition between high- and low-risk groups were assessed, and Spearman correlation analyses were performed to evaluate associations between the risk score and immune cell infiltration. Correlation patterns between model signature genes and immune cell types were also examined to characterize potential links between risk-related molecular features and immune niches.

### 2.9 Somatic mutation analysis

At the genomic level, somatic mutation data from TCGA-LUAD were used to generate mutational landscape summaries for the high- and low-risk groups. Co-occurrence and mutual exclusivity analyses were conducted for frequently mutated genes to compare mutational features and combinations of genomic events across risk states.

### 2.10 Drug sensitivity prediction

To explore potential differences in therapeutic response, half-maximal inhibitory concentrations (IC50) for multiple anticancer agents were predicted based on transcriptomic expression features. Wilcoxon rank-sum tests were used to compare predicted IC50 values between high- and low-risk groups, thereby identifying statistically significant candidate drug signals (Sebaugh, 2011).

### 2.11 *In vitro* functional validation of VWA1

To validate the biological function of a key prognostic signature gene, VWA1, which showed the highest feature importance in the random survival forest model, was selected for *in vitro* experiments. The human LUAD cell line A549 was maintained under standard culture conditions. Cells transfected with an empty vector plasmid were used as the negative control group (OE-NC), and cells transfected with a Flag-tagged VWA1 overexpression plasmid constituted the overexpression group (OE-VWA1). After transfection, VWA1 overexpression efficiency was verified by detecting the Flag signal via Western blotting, with GAPDH used as the loading control (Zhong et al., 2023).

Cell proliferation was assessed using the CCK-8 assay. Absorbance at 450 nm was measured at 0, 24, 48, and 72 h, and growth curves were plotted to compare proliferation between OE-NC and OE-VWA1 cells. Cell migration was evaluated using a wound-healing assay. Images were captured at 0 and 24 h after scratching, and changes in relative wound width were calculated to quantify migratory capacity between groups (Han et al., 2024).

To further assess whether VWA1 expression was responsive to hypoxic stimulation, A549 cells were cultured under normoxic and hypoxic conditions. HIF-1α was used as a hypoxia-response control. VWA1 mRNA expression was quantified by qPCR at different time points after hypoxic treatment, and VWA1 protein expression was assessed by Western blotting. GAPDH was used as the loading control. For qPCR analysis, ACTB was used as the internal reference gene, and the primer sequences, product sizes, and melting temperatures are listed in Table 1. All *in vitro* experiments were independently repeated at least three times (Liang et al., 2007).

**TABLE 1:** Primer sequences used for qPCR.

Gene	Forward primer (5′–3′)	Reverse primer (5′–3′)	Product size (bp)	Tm (^°^C)
VWA1	GCT​GAG​AAG​CTG​GAC​AAG​GA	GCTGCTGCTGCTGCTGTA	142	60.0
HIF1A	CAG​AAG​ATC​TCG​GCG​AAG​CA	GTG​GTG​GCA​TGG​TGA​GTT​TG	138	59.8
ACTB	CAT​GTA​CGT​TGC​TAT​CCA​GGC	CTC​CTT​AAT​GTC​ACG​CAC​GAT	250	60.2
GAPDH	GGA​GCG​AGA​TCC​CTC​CAA​AA	GGC​TGT​TGT​CAT​ACT​TCT​CAT​GG	197	59.7

### 2.12 Statistical analysis

All statistical analyses were performed in R (v4.5.1). Continuous variables were compared using the Wilcoxon rank-sum test, and categorical variables were compared using the chi-square test. Correlations were assessed using Spearman’s correlation coefficient. Survival analyses were conducted using the Kaplan-Meier method and the log-rank test. Cox regression was applied to evaluate prognostic factors. For analyses involving multiple comparisons, P values were adjusted using the Benjamini–Hochberg false discovery rate (FDR) procedure where appropriate. Samples lacking key follow-up information were excluded before survival modeling. All tests were two-sided, and P < 0.05 was considered statistically significant.

## 3 Results

**FIGURE 1:**
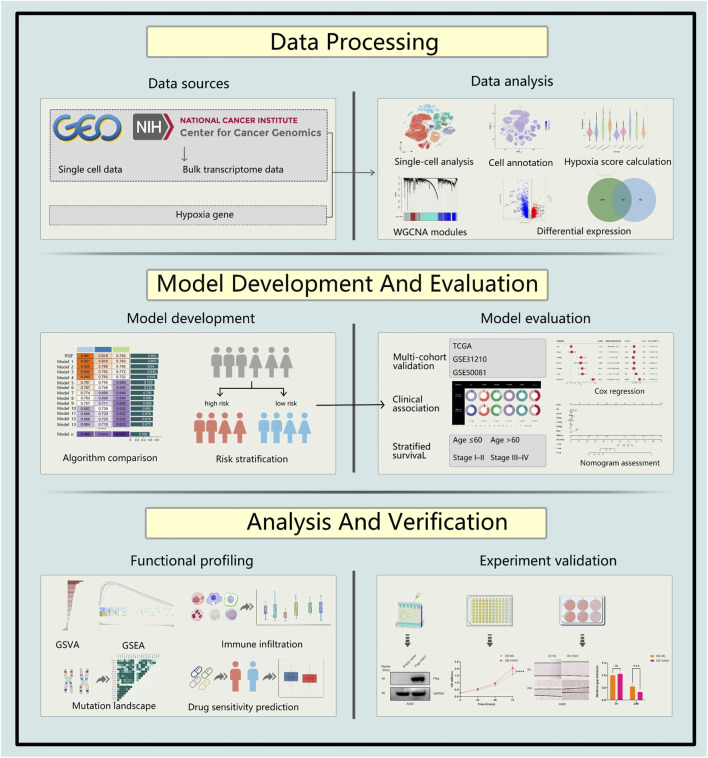
Study flowchart.

All analytical processes are illustrated in the flowchart (Figure 1).

### 3.1 Single-cell atlas delineates the cellular composition and hypoxia heterogeneity of the LUAD tumor microenvironment

**FIGURE 2:**
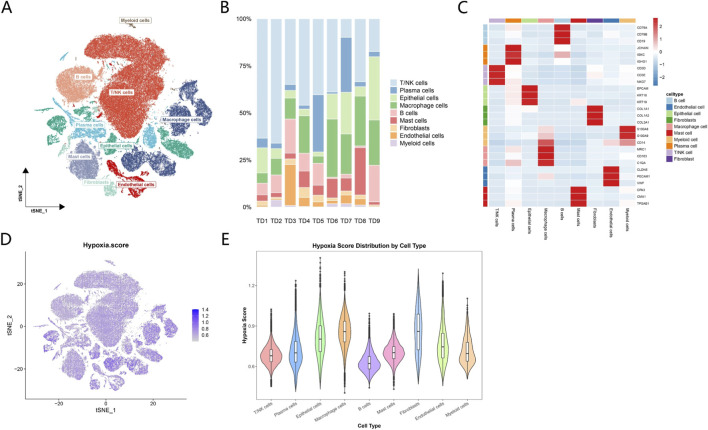
Single-cell landscape of the lung adenocarcinoma tumor microenvironment and distribution of hypoxia features. **(A)** tSNE projection of single cells from lung adenocarcinoma samples, colored by annotated cell types, including T/NK cells, B cells, plasma cells, epithelial cells, macrophage cells, mast cells, fibroblasts, endothelial cells, and myeloid cells. Each dot represents one cell. **(B)** Stacked bar chart showing the relative proportions of major cell types across individual samples (TD1–TD9). **(C)** Heatmap displaying the expression patterns of representative marker genes across different cell types. Rows indicate marker genes and columns represent cell types. Color scale denotes scaled gene expression levels. **(D)** tSNE embedding colored by Hypoxia score at the single-cell level, illustrating the spatial distribution of hypoxia-related transcriptional activity across the tumor microenvironment. **(E)** Violin plot showing the distribution of Hypoxia scores across different cell types. Each dot represents an individual cell. Center line indicates the median, and boxes denote the interquartile range.

To systematically characterize the cellular composition and functional states of the LUAD tumor microenvironment, we performed single-cell transcriptomic profiling on multiple tumor samples. After quality control and batch correction, all cells were subjected to dimensionality reduction and unsupervised clustering. Based on the expression patterns of canonical marker genes, nine major cell populations were identified, including T/NK cells, B cells, plasma cells, epithelial cells, macrophage cells, mast cells, fibroblasts, endothelial cells, and myeloid cells (Figure 2A). The t-SNE projection showed that these populations formed relatively distinct and well-separated clusters in the low-dimensional space, indicating cell-type-specific transcriptional signatures.

Cellular composition differed markedly across tumor samples (Figure 2B). In TD1 and TD2, T/NK cells constituted the predominant fraction, accompanied by appreciable proportions of macrophage cells and epithelial cells. In contrast, TD3 exhibited a notable increase in the proportions of B cells and endothelial cells. TD4 was mainly composed of T/NK cells and macrophage cells, whereas TD5 showed relatively high fractions of T/NK cells and plasma cells. Notably, B cells remained abundant in TD9. Overall, the proportions of immune cells, tumor-associated epithelial cells, and stromal-related cell populations varied substantially across samples, highlighting prominent inter-individual heterogeneity in the LUAD tumor microenvironment.

To validate the accuracy of cell-type annotation, we further visualized the expression of canonical marker genes across cell populations (Figure 2C). T/NK cells highly expressed CD3D, CD3E, and NKG7. B cells showed prominent expression of CD79A, CD79B, and CD19, whereas plasma cells were characterized by enriched expression of JCHAIN, IGKC, and IGHG1. In epithelial cells, EPCAM, KRT18, and KRT19 were markedly upregulated. Fibroblasts primarily expressed COL1A1, COL1A2, and COL3A1, while endothelial cells highly expressed PECAM1, VWF, and CLDN5. Among myeloid-lineage populations, macrophage cells showed high expression of MRC1, CD163, and C1QA, whereas myeloid cells were characterized by S100A8 and S100A9. In contrast, mast cells specifically expressed CPA3, CMA1, and TPSAB1. These marker genes displayed concentrated expression within their corresponding populations and clearly distinguished them from other cell types, supporting the reliability of our annotations.

Given that tumor hypoxia is a key driver of LUAD progression and treatment response, we computed a hypoxia score for each cell using a curated hypoxia-related gene set and mapped the scores onto the t-SNE space (Figure 2D). Overall, hypoxia signaling was not uniformly distributed. Instead, high-score regions were concentrated within specific cell populations and localized areas, suggesting pronounced spatial and cellular-level hypoxia heterogeneity within the tumor microenvironment.

We next compared the distribution of hypoxia scores across cell types (Figure 2E). Macrophage cells and fibroblasts exhibited the highest hypoxia scores overall, with median values clearly exceeding those of other populations. Epithelial cells and endothelial cells displayed intermediate hypoxia scores. In contrast, lymphocyte-related populations, including T/NK cells, plasma cells, and B cells, generally showed low hypoxia scores. Among them, B cells had the lowest hypoxia scores with a relatively narrow distribution. Collectively, these descriptive single-cell results suggest that hypoxia status within the tumor microenvironment is non-uniform at the cellular level.

### 3.2 Identification and characterization of hypoxia-related modules using co-expression network analysis

**FIGURE 3:**
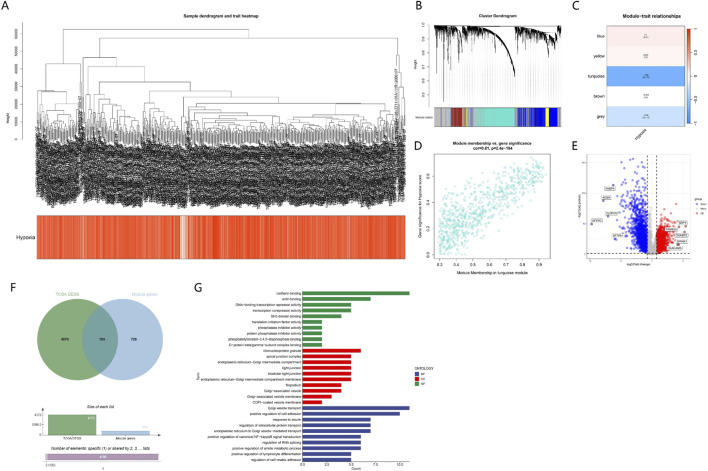
Identification of hypoxia-related gene modules and hub genes using WGCNA. **(A)** Sample dendrogram based on hierarchical clustering of gene expression profiles from the TCGA-LUAD cohort, with Hypoxia score shown as a sample trait heatmap below, illustrating the distribution of hypoxia status across samples. **(B)** Hierarchical clustering dendrogram of genes constructed by WGCNA. Different colors represent distinct gene co-expression modules identified by dynamic tree cutting based on topological overlap. **(C)** Heatmap showing correlations between module eigengenes and Hypoxia score. Colors indicate the direction and strength of correlations, and numbers represent correlation coefficients with corresponding P values. **(D)** Scatter plot illustrating the relationship between gene significance (GS) for hypoxia and module membership (MM) within the turquoise module. Each dot represents a gene. **(E)** Volcano plot displaying differentially expressed genes (DEGs) between the hypoxia group and the control group. Red and blue dots indicate significantly upregulated and downregulated genes, respectively. **(F)** Venn diagram showing the overlap between genes in the turquoise module and differentially expressed genes, identifying 103 hypoxia-related key genes. **(G)** Gene Ontology (GO) enrichment analysis of the 103 key genes, including Biological Process (BP), Cellular Component (CC), and Molecular Function (MF) categories. Bubble size represents the number of enriched genes and color indicates statistical significance.

To identify key gene modules associated with the hypoxia phenotype at the transcriptome-wide level, we constructed a weighted gene co-expression network using the TCGA-LUAD cohort. Hierarchical clustering of samples showed no obvious outliers in global expression patterns, and the hypoxia score displayed a continuous distribution across samples without abrupt changes along dendrogram branches (Figure 3A).

At the gene level, hierarchical clustering coupled with the dynamic tree cutting algorithm identified multiple stable co-expression modules. These modules differed substantially in gene number and internal structure, and each formed a relatively independent branch in the clustering dendrogram (Figure 3B). Correlation analyses between module eigengenes and the hypoxia score revealed marked variability in hypoxia associations across modules. Among them, the turquoise module exhibited the strongest negative correlation with hypoxia (r = −0.66, P = 3e−75), substantially exceeding that of other modules, indicating a tight association with hypoxia status (Figure 3C). Therefore, the turquoise module was selected as the core hypoxia-related module for downstream analyses. Within this module, module membership was strongly and positively correlated with gene significance for hypoxia (cor = 0.81, P = 2.4e−194). The scatter plot showed that many genes simultaneously displayed high module membership and high hypoxia-related significance, suggesting concordance between intramodular connectivity and the strength of association with the hypoxia phenotype (Figure 3D).

In parallel, differential expression analysis was performed between the hypoxia and control groups to identify genes with significant expression changes. The volcano plot illustrated the global distribution of differentially expressed genes (DEGs). Several genes, including SPP1, CRABP2, SPINK1, and CEACAM5, were significantly upregulated in tumor tissues, whereas FABP4, AGER, CLDN18, SFTPA1, and SFTPC were markedly downregulated (Figure 3E). To further refine the candidate set and obtain a stable hypoxia-related gene signature, we intersected the turquoise module genes with the DEGs. The Venn diagram identified 103 overlapping genes, which were defined as hypoxia-related key candidate genes (Figure 3F).

To characterize the functional features of these 103 key genes, Gene Ontology (GO) enrichment analysis was performed (Figure 3G). The enriched terms showed clear functional patterns across categories. In the Biological Process (BP) category, genes were mainly enriched in terms such as Golgi vesicle transport and positive regulation of cell adhesion. In the Cellular Component (CC) category, they were primarily associated with ribonucleoprotein granule and apical junction complex. In the Molecular Function (MF) category, the most significantly enriched terms included cadherin binding and actin binding. Collectively, these results suggest that the hypoxia-related key genes are functionally concentrated in processes related to cell adhesion and intracellular material transport.

### 3.3 Development of a hypoxia-related prognostic model and validation across multiple cohorts

**FIGURE 4:**
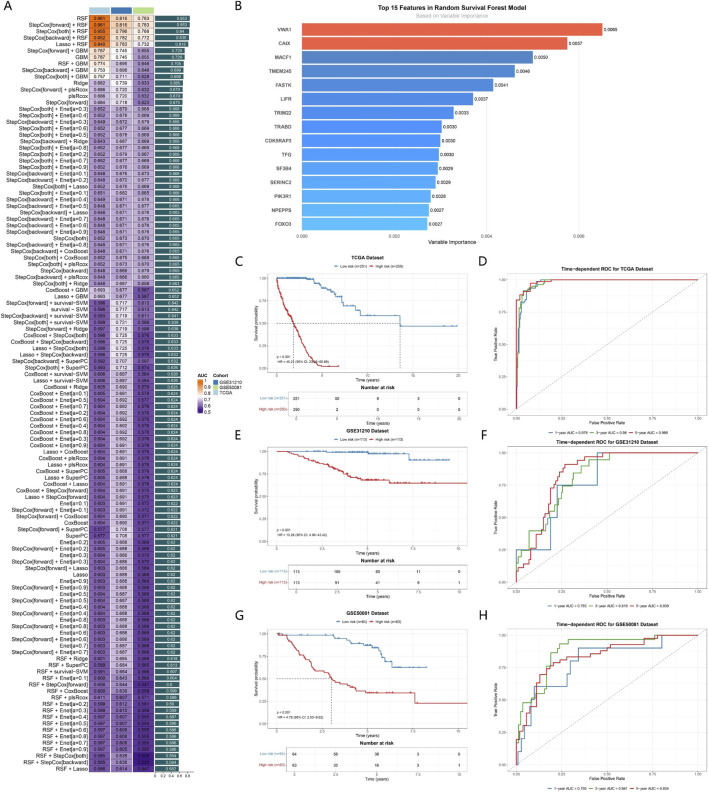
Construction and validation of a hypoxia-related prognostic model based on hub genes. **(A)** Heatmap showing the AUC values of different machine learning and regression model combinations across the TCGA, GSE31210, and GSE50081 cohorts. **(B)** Variable importance ranking of the top 15 genes in the Random Survival Forest (RSF) model. **(C)** Kaplan–Meier survival curves of overall survival for high-risk and low-risk groups in the TCGA cohort stratified by the median risk score. **(D)** Time-dependent ROC curves for predicting 1-, 3-, and 5-year overall survival in the TCGA cohort. **(E)** Kaplan–Meier survival curves of overall survival for high-risk and low-risk groups in the GSE31210 cohort. **(F)** Time-dependent ROC curves for predicting 1-, 3-, and 5-year overall survival in the GSE31210 cohort. **(G)** Kaplan–Meier survival curves of overall survival for high-risk and low-risk groups in the GSE50081 cohort. **(H)** Time-dependent ROC curves for predicting 1-, 3-, and 5-year overall survival in the GSE50081 cohort.

Based on the 103 hypoxia-related key genes, we constructed survival prediction models using multiple machine learning and regression algorithms and compared their performance across the TCGA, GSE31210, and GSE50081 cohorts. The AUC values varied among different algorithmic combinations. Overall, RSF-based strategies showed superior performance across the three cohorts and maintained relatively stable predictive accuracy across datasets (Figure 4A).

Within the RSF model, feature genes with the highest contributions were identified according to variable importance rankings. VWA1 and CAIX exhibited markedly higher importance scores than other variables, and the importance values showed a graded distribution across genes (Figure 4B).

Patients in the TCGA cohort were then stratified into high-risk and low-risk groups according to the median risk score. Kaplan-Meier analysis demonstrated that the high-risk group had significantly poorer overall survival than the low-risk group (P < 0.001) (Figure 4C). Time-dependent ROC analysis further evaluated the model’s predictive performance at different time points, yielding AUC values of 0.978, 0.980, and 0.989 for 1-year, 3-year, and 5-year overall survival, respectively (Figure 4D), indicating strong discriminative capability.

To assess generalizability, the same risk score formula was applied to two independent external cohorts, GSE31210 and GSE50081. In the GSE31210 cohort, the survival curves of the high-risk and low-risk groups were also clearly separated (P < 0.001) (Figure 4E). The corresponding time-dependent ROC curves showed AUC values of 0.785, 0.818, and 0.839 for 1-year, 3-year, and 5-year overall survival, respectively (Figure 4F). Consistent results were obtained in the GSE50081 cohort. After stratification by the risk score, patients in the high-risk group exhibited significantly lower survival probabilities than those in the low-risk group (P < 0.001) (Figure 4G). Time-dependent ROC analysis yielded AUC values of 0.795, 0.881, and 0.834 for 1-year, 3-year, and 5-year overall survival, respectively (Figure 4H).

### 3.4 Model robustness diagnostics

To address concerns regarding optimism and overfitting, two quantitative diagnostics were performed. First, optimism-corrected internal validation using 1,000 bootstrap resamples yielded a corrected C-index of 0.847 (95% CI: 0.827–0.878) and corrected 1/3/5-year AUCs of 0.874, 0.897, and 0.897, respectively (Table 2). The estimated optimism (∼0.10) confirms that the apparent training performance was inflated; however, the corrected estimates remain within the range observed in the external validation cohorts (C-index 0.78–0.88), supporting the consistency of the model’s discrimination across resampling regimes. Second, calibration analysis at 1, 3, and 5 years (Table 3) revealed acceptable overall calibration (O/E ratio 0.91–0.97 in TCGA; 0.55–0.84 in GSE50081), although calibration slopes deviated from unity, consistent with the well-documented tendency of random survival forests to produce shrunken absolute probability estimates. The lower O/E ratios observed in GSE31210 reflect the predominantly early-stage composition of that cohort and suggest that absolute risk recalibration—rather than re-derivation of the risk score—would be required prior to clinical deployment.

**TABLE 2:** Optimism-corrected internal performance (1,000 bootstrap resamples).

Metric	Apparent	Optimism	Corrected	95% bootstrap CI
C-index	0.961	0.114	0.847	0.827–0.878
1-year AUC	0.985	0.111	0.874	0.832–0.913
3-year AUC	0.991	0.094	0.897	0.877–0.926
5-year AUC	0.996	0.099	0.897	0.866–0.925

**TABLE 3:** Calibration performance.

Cohort	Time	Slope (95% CI)	O/E ratio	Observed	Expected
TCGA	1 years/3 years/5 years	3.43 (3.09–3.76)/3.77/3.22	0.91/0.94/0.97	0.13/0.30/0.44	0.15/0.32/0.45
GSE31210	1 years/3 years/5 years	1.49/2.27/2.45	0.14/0.33/0.41	0.02/0.09/0.16	0.13/0.28/0.39
GSE50081	1 years/3 years/5 years	1.36/1.84/1.80	0.55/0.75/0.84	0.08/0.25/0.39	0.15/0.33/0.46

### 3.5 Associations between the risk score and clinicopathological features and stratified survival analyses

**FIGURE 5:**
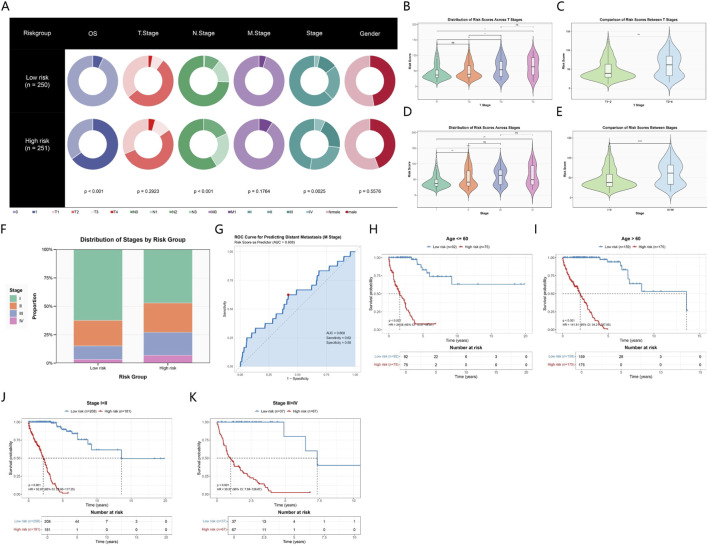
Association between hypoxia-related risk score and clinical characteristics and stratified survival analysis. **(A)** Distribution of overall survival status (OS), T stage, N stage, M stage, clinical stage, and gender between low-risk and high-risk groups in the TCGA cohort. **(B)** Distribution of risk scores across different T stages. **(C)** Comparison of risk scores between T1–2 and T3–4 groups. **(D)** Distribution of risk scores across different clinical stages (Stage I–IV). **(E)** Comparison of risk scores between Stage I–II and Stage III–IV groups. **(F)** Proportional distribution of clinical stages in the low-risk and high-risk groups. **(G)** ROC curve of the risk score for predicting distant metastasis (M stage). **(H)** Kaplan-Meier survival curves of overall survival for low-risk and high-risk groups in patients aged ≤60 years. **(I)** Kaplan-Meier survival curves of overall survival for low-risk and high-risk groups in patients aged >60 years. **(J)** Kaplan-Meier survival curves of overall survival for low-risk and high-risk groups in patients with Stage I–II disease. **(K)** Kaplan-Meier survival curves of overall survival for low-risk and high-risk groups in patients with Stage III–IV disease. *P < 0.05, **P < 0.01, ***P < 0.001.

To evaluate the relationships between the hypoxia-related risk score and clinicopathological characteristics, patients in the TCGA cohort were stratified into high-risk and low-risk groups using the median risk score. Distributions of multiple clinical variables were then compared between groups (Figure 5A). Significant differences were observed in OS (P < 0.001), with a markedly higher proportion of deaths in the high-risk group. N stage also differed significantly between groups (P < 0.001), and a greater fraction of patients with advanced N stage was observed in the high-risk group. Clinical stage (Stage) likewise showed a statistically significant difference (P = 0.0025), with a higher proportion of late-stage cases in the high-risk group. In contrast, no significant differences were detected for T stage, M stage, or gender between high-risk and low-risk groups (all P > 0.05).

We next examined the distribution of risk scores across different T stages. The risk score showed an increasing trend with higher T stage, and patients with T3–T4 disease exhibited significantly higher risk scores than those with T1–T2 disease (Figures 5B,C). A similar pattern was observed across clinical stages, with patients in Stage III–IV showing overall higher risk scores than those in Stage I–II (Figures 5D,E).

We further compared the composition of clinical stages between the high-risk and low-risk groups. The high-risk group contained a markedly higher proportion of Stage III and Stage IV patients, whereas the low-risk group was predominantly composed of Stage I and Stage II patients (Figure 5F). To assess the ability of the risk score to discriminate distant metastasis, we constructed an ROC curve using M stage as the outcome. The risk score demonstrated modest discriminatory performance for predicting distant metastasis, with an AUC of 0.608 (Figure 5G).

Stratified survival analyses were then performed across clinical subgroups. In both age subgroups (≤60 years and >60 years), patients in the high-risk group had significantly poorer overall survival than those in the low-risk group (both P < 0.001) (Figures 5H,I). In the Stage I–II subgroup, overall survival was markedly lower in the high-risk group than in the low-risk group (P < 0.001) (Figure 5J). The same trend was observed in the Stage III–IV subgroup, where the high-risk group also exhibited significantly reduced overall survival (P < 0.001) (Figure 5K).

### 3.6 Identification of independent prognostic factors and construction and evaluation of a nomogram

**FIGURE 6:**
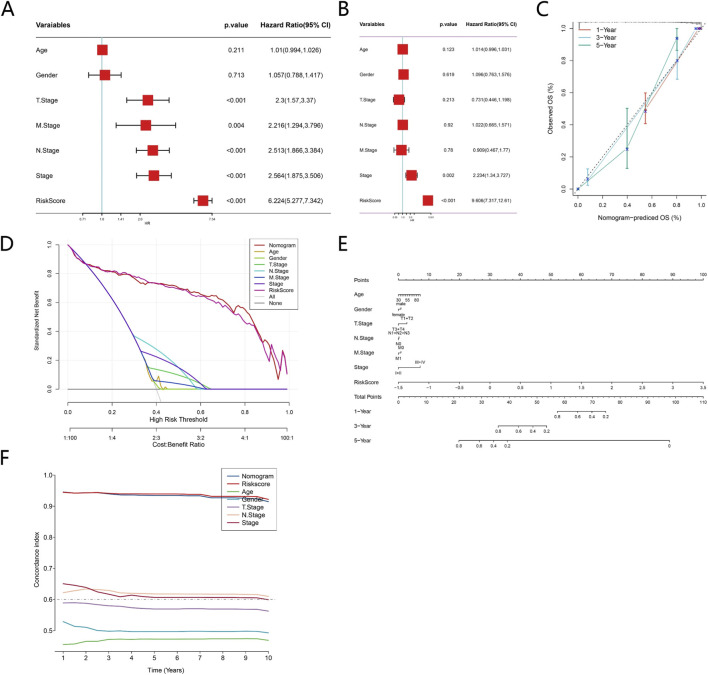
Independent prognostic analysis and construction of a nomogram model integrating the hypoxia-related risk score and clinical variables. **(A)** Univariate Cox regression analysis of overall survival for clinical variables and the hypoxia-related risk score in the TCGA cohort. **(B)** Multivariate Cox regression analysis identifying independent prognostic factors for overall survival in the TCGA cohort. **(C)** Calibration curves of the nomogram for predicting 1-, 3-, and 5-year overall survival, showing the agreement between predicted and observed outcomes. **(D)** Decision curve analysis (DCA) evaluating the clinical net benefit of the nomogram model compared with other predictive models across a range of threshold probabilities. **(E)** Nomogram constructed based on independent prognostic factors to predict 1-, 3-, and 5-year overall survival probabilities for patients with lung adenocarcinoma. **(F)** Time-dependent concordance index (C-index) comparison of different models during follow-up, including the nomogram model, risk score model, and individual clinical variables.

Univariate Cox regression analysis was first performed in the TCGA cohort to evaluate associations between clinicopathological variables, the risk score, and overall survival (Figure 6A). T stage, N stage, M stage, clinical stage, and the risk score were all significantly associated with overall survival (all P < 0.01), whereas age and gender were not statistically significant. These variables were then entered into a multivariate Cox regression model (Figure 6B). After adjustment for other clinical factors, the risk score remained a significant independent predictor (P < 0.001), and clinical stage also remained significantly associated with overall survival, while the effects of other variables were attenuated.

Calibration curves were used to assess the agreement between predicted and observed survival probabilities at different time points (Figure 6C). The calibration curves for 1-, 3-, and 5-year survival closely approximated the ideal reference line, indicating good predictive consistency. Decision curve analysis was conducted to evaluate the clinical net benefit of the nomogram across a range of threshold probabilities and to compare it with single-variable models (Figure 6D). The nomogram yielded higher net benefit over a broad range of threshold probabilities, suggesting improved potential clinical utility.

Based on independent prognostic factors identified by multivariate Cox regression, a nomogram was constructed to predict 1-, 3-, and 5-year overall survival probabilities (Figure 6E). Each variable was assigned a point value proportional to its regression coefficient, and the total points were used to generate individualized survival predictions. Finally, time-dependent concordance index analyses were performed to compare discriminative ability of different models during follow-up (Figure 6F). The nomogram showed consistently higher C-index values than models using the risk score alone or traditional clinical variables, indicating more stable predictive performance over time.

### 3.7 Functional enrichment analyses and comparison of hypoxia-related pathway features

**FIGURE 7:**
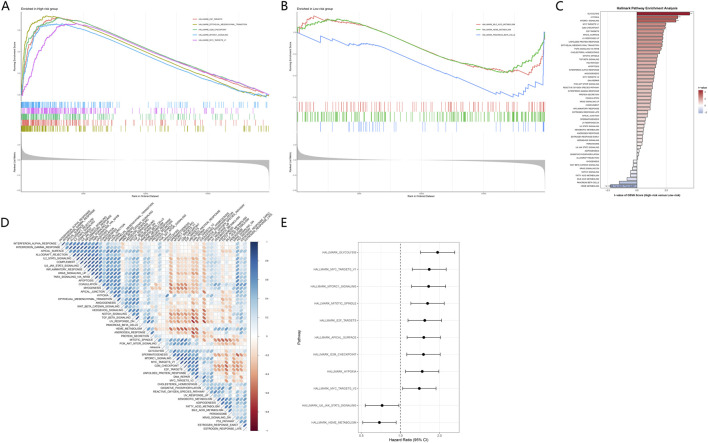
Functional enrichment analysis of hypoxia-related risk groups based on Hallmark pathways. **(A)** Gene set enrichment analysis (GSEA) showing Hallmark pathways significantly enriched in the high-risk group. **(B)** Gene set enrichment analysis (GSEA) showing Hallmark pathways significantly enriched in the low-risk group. **(C)** Hallmark pathway enrichment analysis based on GSVA scores comparing pathway activities between the high-risk and low-risk groups. **(D)** Heatmap displaying the GSVA scores of Hallmark pathways across individual samples, illustrating distinct functional patterns between the two risk groups. **(E)** Forest plot showing the results of univariate Cox regression analysis for Hallmark pathways associated with overall survival.

To investigate biological functional differences between the high-risk and low-risk groups, GSEA was performed based on gene expression profiles. In the high-risk group, multiple tumor-related signaling pathways were significantly enriched, including HALLMARK_E2F_TARGETS, HALLMARK_EPITHELIAL_MESENCHYMAL_TRANSITION, HALLMARK_G2M_CHECKPOINT, HALLMARK_MTORC1_SIGNALING, and HALLMARK_MYC_TARGETS_V1 (Figure 7A). In contrast, the low-risk group was mainly enriched in metabolism- and homeostasis-related pathways, including HALLMARK_BILE_ACID_METABOLISM, HALLMARK_HEME_METABOLISM, and HALLMARK_PANCREAS_BETA_CELLS (Figure 7B), indicating clear metabolic differences between the two groups.

We further performed GSVA-based differential analysis of Hallmark pathways to systematically assess global changes in pathway activity between the high-risk and low-risk groups (Figure 7C). In the high-risk group, the pathways with the highest GSVA scores mainly included HALLMARK_GLYCOLYSIS, HALLMARK_HYPOXIA, and HALLMARK_MTORC1_SIGNALING. By contrast, HALLMARK_HEME_METABOLISM showed a significant negative association with the high-risk group and exhibited higher pathway activity in the low-risk group. These findings indicate marked differences in metabolic and hypoxia-related pathway activity between the two risk groups. To visualize pathway activity patterns across individual samples, a heatmap of Hallmark pathway activity was generated (Figure 7D). Distinct clustering patterns were observed between high-risk and low-risk groups, suggesting consistent functional divergence associated with risk stratification.

Univariate Cox regression analysis was further applied to evaluate associations between Hallmark pathways and overall survival (Figure 7E). Multiple pathways were significantly associated with overall survival. Among them, HALLMARK_GLYCOLYSIS, HALLMARK_MYC_TARGETS_V1, HALLMARK_MTORC1_SIGNALING, and HALLMARK_MITOTIC_SPINDLE showed hazard ratios greater than 1, whereas HALLMARK_HEME_METABOLISM showed an opposite trend.

### 3.8 Immune pathway activity and immune cell infiltration characteristics across risk groups

**FIGURE 8:**
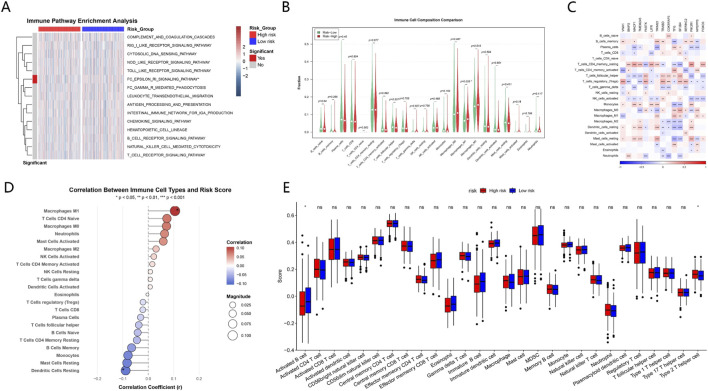
Immune pathway activity and immune cell infiltration characteristics between high-risk and low-risk groups. **(A)** ssGSEA-based immune pathway activity analysis comparing high-risk and low-risk groups. **(B)** CIBERSORT-based comparison of immune cell composition between high-risk and low-risk groups. **(C)** Heatmap showing correlations between model feature genes and CIBERSORT-estimated immune cell types. **(D)** Correlation analysis between CIBERSORT-estimated immune cell infiltration levels and risk score. Macrophages M1 shows a positive correlation with risk score, whereas Dendritic Cells Resting shows a negative correlation. **(E)** Boxplots comparing CIBERSORT-estimated immune cell infiltration levels between high-risk and low-risk groups.

To further investigate differences in immune-related functions between the high-risk and low-risk groups, immune pathway enrichment analysis was performed (Figure 8A). Among the analyzed immune-related pathways, only the FC_EPSILON_RI_SIGNALING_PATHWAY showed a statistically significant difference between the high-risk and low-risk groups, whereas no significant changes were observed for the remaining pathways. These findings suggest that immune pathway differences between the two groups were relatively limited and mainly concentrated in selected immune-related signals.

We next compared immune cell composition between the high-risk and low-risk groups (Figure 8B). Among all immune cell subsets, only Macrophages_M1 showed a significant difference between groups (P = 0.028), whereas differences in infiltration levels of other immune cell types did not reach statistical significance.

We then analyzed correlations between model feature genes and immune cell types (Figure 8C). Most feature genes showed varying degrees of correlation with multiple immune cell subsets, and distinct immune cell types exhibited different correlation patterns, indicating potential links between risk model-related genes and the immune microenvironment. Correlation analysis was further performed to evaluate associations between immune cell infiltration levels and the risk score (Figure 8D). Macrophages M1 was significantly positively correlated with the risk score, whereas resting dendritic cells were significantly negatively correlated with the risk score. No significant correlations were observed between the risk score and other immune cell types.

To further compare immune cell infiltration differences between the high-risk and low-risk groups, boxplots were generated for quantitative analysis (Figure 8E). Only activated B cells and type 2 T helper cells showed statistically significant differences between groups, whereas no significant differences were observed for the remaining immune cell subsets.

### 3.9 Comparison of somatic mutation landscape characteristics between high-risk and low-risk groups

**FIGURE 9:**
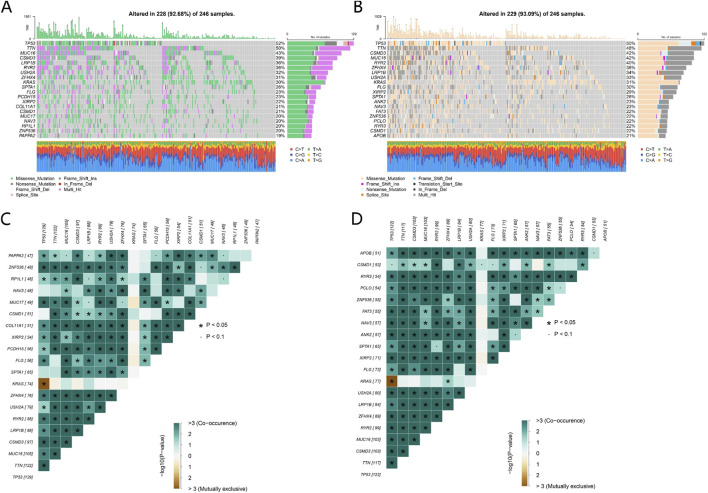
Somatic mutation landscape in the high-risk and low-risk groups analyzed using maftools. **(A)** Waterfall plot showing the somatic mutation profile of the high-risk group. **(B)** Waterfall plot showing the somatic mutation profile of the low-risk group. **(C)** Co-occurrence and mutual exclusivity analysis of frequently mutated genes in the high-risk group. **(D)** Co-occurrence and mutual exclusivity analysis of frequently mutated genes in the low-risk group.

To compare genomic mutation patterns between the high-risk and low-risk groups, somatic mutation data were systematically analyzed and visualized using waterfall plots. In the high-risk group, gene mutation events were detected in 228 of 246 samples (92.68%) (Figure 9A), whereas 229 of 246 samples (93.09%) harbored mutations in the low-risk group (Figure 9B), indicating comparable overall mutation frequencies between groups. In the high-risk group, the most frequently mutated genes included TP53, TTN, MUC16, and CSMD3. In the low-risk group, high-frequency mutations were likewise dominated by TP53, TTN, CSMD3, and MUC16, suggesting substantial similarity in the major mutational composition between the two groups.

We further analyzed co-occurrence and mutual exclusivity patterns among mutated genes in the high-risk group (Figure 9C). Most gene pairs showed significant co-occurrence, whereas KRAS exhibited mutual exclusivity trends with several gene pairs. In the low-risk group, co-occurrence relationships also predominated among mutated genes (Figure 9D), and mutual exclusivity trends involving KRAS and several gene pairs were likewise observed. Overall, the high-risk and low-risk groups showed partially consistent correlation patterns among mutated genes, although the strengths of associations differed for specific gene pairs.

### 3.10 Drug sensitivity differences between high-risk and low-risk groups

**FIGURE 10:**
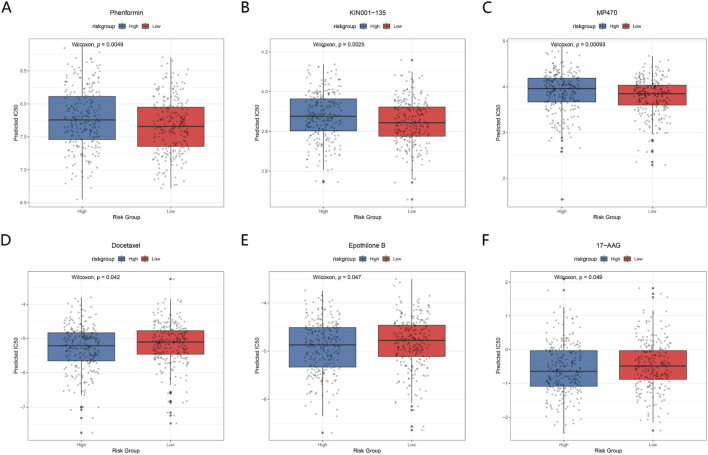
Drug sensitivity differences between high-risk and low-risk groups. **(A)** Predicted IC50 of Phenformin between the high-risk and low-risk groups. **(B)** Predicted IC50 of KIN001-135 between the high-risk and low-risk groups. **(C)** Predicted IC50 of MP470 between the high-risk and low-risk groups. **(D)** Predicted IC50 of Docetaxel between the high-risk and low-risk groups. **(E)** Predicted IC50 of Epothilone B between the high-risk and low-risk groups. **(F)** Predicted IC50 of 17-AAG between the high-risk and low-risk groups.

To explore drug sensitivity patterns associated with the hypoxia-related risk model, we used the pRRophetic algorithm to predict the half-maximal inhibitory concentration (IC50) of multiple anticancer drugs in the high-risk and low-risk groups. The analysis revealed significant heterogeneity in drug response profiles between the two groups (Figure 10).

First, for several metabolic modulators and kinase inhibitors, the high-risk group exhibited potential drug resistance features. Specifically, the predicted IC50 values of phenformin (Figure 10A, P = 0.0049), KIN001-135 (Figure 10B, P = 0.0025), and MP470 (Figure 10C, P = 0.00093) were significantly higher in the high-risk group than in the low-risk group. These findings suggest that patients in the low-risk group may be more likely to benefit from these agents.

In contrast, although patients in the high-risk group generally had poorer prognosis, drug sensitivity analysis indicated greater sensitivity to specific chemotherapeutic and targeted agents, as reflected by lower predicted IC50 values. As shown in Figures 10D–F, the microtubule-stabilizing agents docetaxel (Figure 10D, P = 0.042) and epothilone B (Figure 10E, P = 0.047), as well as the HSP90 inhibitor 17-AAG (Figure 10F, P = 0.049), all showed significantly lower predicted IC50 values in the high-risk group. These findings indicate that the high-risk subgroup may have distinct predicted sensitivity profiles for selected cytotoxic and targeted agents.

### 3.11 *In vitro* functional validation of the key feature gene VWA1

**FIGURE 11:**
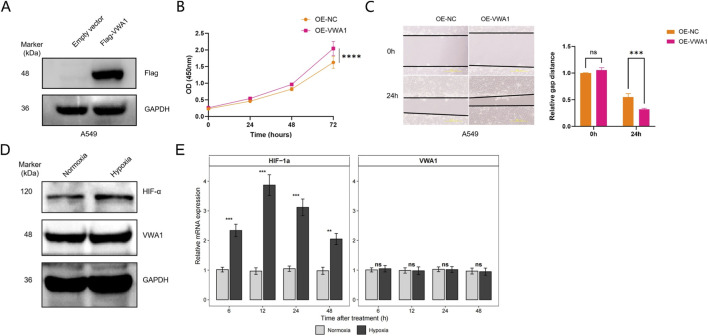
*In vitro* functional validation and hypoxia-response assessment of VWA1 in lung adenocarcinoma cells. **(A)** Western blot analysis confirming VWA1 overexpression in A549 cells transfected with Empty vector (OE-NC) or Flag-tagged VWA1 plasmid (OE-VWA1), with GAPDH as the loading control. **(B)** CCK-8 assay showing cell proliferation curves of OE-NC and OE-VWA1 A549 cells at 0, 24, 48, and 72 h. **(C)** Wound-healing assay assessing the migratory capacity of OE-NC and OE-VWA1 cells, with representative images at 0 h and 24 h and quantification of relative gap distance. **(D)** Western blot analysis of HIF-1α and VWA1 protein expression in A549 cells under normoxic and hypoxic conditions. **(E)** qPCR analysis of HIF-1α and VWA1 mRNA expression after hypoxic stimulation. Data are presented as mean ± SD. Statistical significance was assessed using Student’s t-test. ns, not significant; ***P < 0.001; ****P < 0.0001.

Given that VWA1 had the highest feature importance score in the Random Survival Forest prognostic model, we further investigated its biological function in LUAD through *in vitro* experiments. First, A549 human LUAD cells were transfected with an empty vector (Empty vector/OE-NC) or a Flag-tagged VWA1 overexpression plasmid (Flag-VWA1). Western blot results confirmed that, compared with the empty vector control group, the Flag-VWA1 group stably expressed the target protein, indicating successful establishment of the VWA1 overexpression model in A549 cells (Figure 11A).

To evaluate the effect of VWA1 on cell proliferation, absorbance values (OD at 450 nm) were measured at 0, 24, 48, and 72 h. The cell growth curves showed that, compared with the control group (OE-NC), the VWA1 overexpression group (OE-VWA1) exhibited a markedly increased proliferation rate, with an extremely significant pro-proliferative effect observed at 72 h (P < 0.0001) (Figure 11B).

In addition, a wound-healing assay was performed to assess the regulatory effect of VWA1 on cell migratory capacity. At 24 h after scratching, the relative gap distance in the VWA1 overexpression group was significantly smaller than that in the control group (P < 0.001) (Figure 11C). This phenotypic change confirms that elevated VWA1 expression significantly enhances the migratory and motile capacity of A549 cells. Taken together, these *in vitro* findings demonstrate that overexpression of the key prognostic gene VWA1 significantly promotes LUAD cell proliferation and migration, further supporting its potential pro-tumorigenic role as a high-risk factor at the cellular level.

To further determine whether VWA1 was responsive to hypoxic stimulation, A549 cells were cultured under normoxic and hypoxic conditions. HIF-1α was included as a positive indicator of hypoxic response. Western blot analysis showed that hypoxic treatment increased HIF-1α expression, confirming activation of the hypoxia-response program. However, VWA1 protein expression did not show obvious hypoxia-induced upregulation under the same conditions (Figure 11D). Consistently, qPCR analysis showed that HIF-1α mRNA expression was significantly increased after hypoxic stimulation, whereas VWA1 mRNA expression showed no significant increase at the examined time points (Figure 11E). These results suggest that VWA1 was not directly induced by hypoxia in A549 cells under the tested experimental conditions.

## 4 Discussion

In this study, we integrated single-cell transcriptomic data with multi-cohort bulk transcriptomic datasets to construct a prognostic stratification framework associated with hypoxia-related transcriptional activity in LUAD. Although several hypoxia-related prognostic signatures have been reported previously, many of them were developed primarily from bulk transcriptomic data and therefore could not directly characterize the cellular distribution of hypoxia activity within the tumor microenvironment. Our study addresses this limitation by first mapping hypoxia-related transcriptional activity at single-cell resolution and then using bulk cohorts with clinical follow-up to develop and validate a prognostic model. The single-cell analysis showed that hypoxia signaling was not a homogeneous property of tumor tissues but exhibited marked heterogeneity across cellular populations. Hypoxia scores were highest in macrophage cells and fibroblasts, followed by epithelial cells and endothelial cells, whereas lymphocyte-related populations showed overall lower scores. This cell-level pattern provides biological context for interpreting inter-patient variation in bulk hypoxia-related signals and supports the rationale for developing a hypoxia-centered risk stratification model (Li et al., 2021).

Based on this phenotype-anchored strategy, WGCNA identified co-expression modules significantly associated with hypoxia in the TCGA-LUAD cohort, and cross-validation with differential expression results between the hypoxia and control groups ultimately yielded 103 key candidate genes. Compared with modeling strategies that start directly from predefined gene sets or a single regularized regression approach, this “module correlation plus differential consistency” screening pipeline places greater emphasis on stable coupling between candidate genes and the hypoxia phenotype, which may help reduce uncertainty arising from feature drift across platforms and cohorts (Johnson et al., 2007). After risk score construction using a Random Survival Forest model, the model consistently stratified patient subgroups with significantly different prognoses in the TCGA training cohort and two independent external cohorts (GSE31210 and GSE50081). We recognize that the time-dependent AUCs in the TCGA training cohort were very high, which may partly reflect optimism associated with model development in the training set. Therefore, we placed greater emphasis on the results from the two independent external validation cohorts when evaluating model generalizability. Although the AUCs in GSE31210 and GSE50081 were lower than those in TCGA-LUAD, they remained within an acceptable range and supported the cross-cohort robustness of the hypoxia-related risk score. Nevertheless, further validation in prospective cohorts and clinically homogeneous datasets is still required before clinical application. The high ranking of CAIX in feature importance is also biologically plausible. As a canonical downstream marker of hypoxia, CAIX has been repeatedly reported to be associated with hypoxia adaptation, poor prognosis, and treatment resistance in LUAD (Koh et al., 2019; Levallet et al., 2023; Fusco et al., 2025). Notably, VWA1 showed the highest feature importance score in our model. To provide functional support for this model-prioritized gene, we performed *in vitro* experiments focusing on VWA1. The results showed that VWA1 overexpression promoted proliferation and migration of A549 LUAD cells, suggesting that VWA1 may contribute to malignant cellular phenotypes and support its potential relevance as a risk-associated gene in the hypoxia-related prognostic signature. We further examined whether VWA1 expression was responsive to hypoxic stimulation. HIF-1α was increased under hypoxic conditions, confirming activation of the hypoxia-response program, whereas VWA1 mRNA and protein expression did not show significant hypoxia-induced upregulation. These findings indicate that VWA1 should not be interpreted as a direct hypoxia-responsive downstream gene in A549 cells under the tested conditions. Rather, VWA1 may represent a prognostically relevant model-prioritized gene within a hypoxia-related transcriptional framework and may contribute to malignant phenotypes through mechanisms not solely dependent on direct hypoxic induction.

Clinical association analyses further demonstrated a systematic concordance between the risk score and disease progression phenotypes. Significant differences between high-risk and low-risk groups were observed in survival status, N stage, and overall clinical stage, with the high-risk group showing a higher proportion of deaths and a greater tendency toward advanced N stage and later clinical stage. The risk score increased with T stage and overall stage, and it stably stratified overall survival across different age groups and clinical stage subgroups. Multivariable Cox regression showed that the risk score retained independent prognostic value after adjustment for clinical variables. Moreover, a nomogram integrating the risk score with clinical variables outperformed single-variable models in calibration, follow-up discrimination, and clinical net benefit (Liang et al., 2015; Balachandran et al., 2015; Vickers and Elkin, 2006). Overall, these findings support the view that hypoxia-related molecular features do not merely recapitulate staging information, but provide an independent dimension with complementary value for clinical stratification.

Pathway-level differences suggest that the high-risk state is not simply characterized by enhanced hypoxia signaling, but rather by an integrated transcriptional program more consistent with an aggressive tumor ecosystem. Enrichment analyses showed that the high-risk group was dominated by cell-cycle and proliferation-driven programs, including E2F targets, G2M checkpoint, and MYC targets, together with enhanced mTORC1 signaling and EMT. In contrast, the low-risk group was more characterized by metabolic homeostasis-related features, with stronger enrichment of bile acid metabolism, heme metabolism, and pancreas beta cells-associated programs. Differences in pathway activity further showed increased glycolysis, hypoxia response, and mTORC1 signaling in the high-risk group, whereas heme metabolism was negatively associated with risk and was more active in the low-risk group. Consistency analyses with survival outcomes supported the same direction. Programs such as glycolysis, MYC targets, mTORC1 signaling, and mitotic spindle were associated with higher mortality risk, whereas heme metabolism showed the opposite trend, suggesting that the high-risk state may be jointly driven by proliferation dependence and metabolic adaptation (Vander Heiden et al., 2009).

Immune analyses revealed a relatively focused pattern of differences. Instead of broad remodeling across immune pathways, the differences were concentrated in specific immune axes and a limited number of cell subsets. At the pathway level, only FC_EPSILON_RI_SIGNALING_PATHWAY reached statistical significance. At the immune infiltration level, the most notable finding was the significant enrichment of M1 macrophages in the high-risk group, together with a positive correlation between M1 macrophages and the risk score. Although M1 macrophages are traditionally considered anti-tumorigenic, their abnormal enrichment in the high-risk context may reflect chronic, unresolved inflammation. Such persistent inflammatory responses may fail to effectively eliminate tumors and instead promote tumor progression through tissue repair mechanisms and stromal remodeling (Kovaleva et al., 2025; Parisi et al., 2018). Meanwhile, resting dendritic cells showed a significant negative correlation with the risk score, suggesting potential impairment of antigen presentation. In addition, differences in activated B cells and type 2 T helper cells between groups further support the complexity of the microenvironment. Collectively, these findings suggest that immune differences associated with the hypoxia-related risk score were relatively focused rather than global. The observed associations with M1 macrophages and resting dendritic cells may reflect selected changes in myeloid-related and antigen-presenting cell-related components of the tumor microenvironment, although further experimental validation is required. Unlike the broad T-cell exhaustion or infiltration patterns commonly reported in previous LUAD studies, the immune alterations identified here were more concentrated, implying that future mechanistic validation should prioritize key myeloid subsets (e.g., macrophage polarization states) and the specific inflammatory microenvironments they mediate, rather than broadly discussing global immune strength (Lian et al., 2024; Wang et al., 2024).

At the genomic level, the overall mutation frequencies were similar between high-risk and low-risk groups, but combinatorial patterns of mutated genes differed. Both groups were characterized by high-frequency mutations in TP53, TTN, MUC16, CSMD3, LRP1B, and KRAS, which is consistent with common LUAD mutational spectra. Co-occurrence and mutual exclusivity analyses suggested mutual exclusivity trends between KRAS and several gene pairs, whereas most other combinations showed co-occurrence. Bristow et al. suggested that the hypoxic microenvironment alters mutational selection pressure by driving genomic instability and altering DNA damage repair pathways (Bristow and Hill, 2008). In our risk stratification framework, the observed differences in mutational combination patterns provide phenomenological evidence that hypoxia-related transcriptional states may be coupled with combinations of genomic events. However, more refined validation incorporating tumor mutational burden (TMB), copy number variation, and driver gene subtypes is still needed.

Finally, drug sensitivity prediction provided pharmacogenomic clues for understanding potential treatment-response differences between risk strata. Interestingly, although the high-risk group showed relative resistance to metabolic modulators such as phenformin (higher predicted IC50 values), it showed significantly greater sensitivity (lower predicted IC50 values) to anti-mitotic agents (e.g., docetaxel and epothilone B) and the HSP90 inhibitor 17-AAG. This observation is highly consistent with our functional enrichment analyses. High-risk tumors were characterized by hyperactive cell-cycle and proliferation signaling (e.g., E2F targets and G2M checkpoint). Because microtubule-stabilizing agents mainly target rapidly dividing cells, this highly proliferative state may render high-risk tumors more vulnerable to these cytotoxic drugs. In addition, hypoxic tumors often experience proteotoxic stress and may be more dependent on molecular chaperones such as HSP90 for survival, which may explain their sensitivity to 17-AAG (Whitesell and Lindquist, 2005; Isaacs et al., 2002). These findings suggest that although high-risk patients have poorer natural prognosis, they may also exhibit distinct predicted sensitivity patterns to selected anti-mitotic agents and HSP90 inhibition, which is consistent with the proliferative and stress-adaptive features of the high-risk state.

Several limitations should be noted. First, the single-cell dataset had a limited sample size and lacked spatial information, making it difficult to directly characterize tissue-level spatial gradients of hypoxia signaling and local niche architecture. In addition, although single-cell-derived hypoxia-associated epithelial genes were used to guide bulk-level candidate gene screening, the final prognostic model did not directly incorporate cell-type deconvolution weights or intercellular communication features. Second, although discrimination of the RSF model was preserved across cohorts, calibration slopes substantially greater than 1 in TCGA and substantially less than 1 in GSE31210/GSE500812 indicate that the absolute predicted probabilities are not directly transferable across platforms. Future external implementation should incorporate a Cox-based recalibration step on the linear predictor before generating individualized survival estimates. Third, although we preliminarily confirmed the pro-proliferative and pro-migratory effects of VWA1 through *in vitro* overexpression experiments, additional mechanistic validation under hypoxia-related experimental conditions is still needed. The precise mechanisms of other model components, as well as conclusions related to immune infiltration and drug sensitivity, also require further functional experiments and clinical confirmation. Nevertheless, starting from single-cell evidence, this study established a hypoxia-related risk stratification system that is complementary to clinical staging and stable across cohorts through network-based screening and machine learning modeling. We further linked this system to proliferative and metabolic programs, immune cell composition, mutational combination patterns, and potential therapeutic differences, providing a clear and testable path for precision stratification and translational research in LUAD.

## 5 Conclusion

This study revealed cellular heterogeneity of hypoxia signaling in LUAD at the single-cell level and established a prognostic risk model based on hypoxia-related key genes. The model achieved stable survival stratification in the TCGA-LUAD, GSE31210, and GSE50081 cohorts and was significantly associated with clinical stage. Functional analyses indicated that the high-risk state was accompanied by enhanced proliferation-driven and metabolic adaptation programs, together with differences in immune features, mutational patterns, and drug sensitivity. *In vitro* experiments further confirmed that VWA1 overexpression promotes proliferation and migration of LUAD cells, supporting its role as a key risk gene. Overall, this study provides a hypoxia-related framework for LUAD risk assessment and offers preliminary clues for future individualized treatment exploration.

## Data Availability

The original contributions presented in the study are included in the article/Supplementary Material, further inquiries can be directed to the corresponding author.
