# Correction: Single-cell dissection and multi-cohort validation identify a hypoxia-related prognostic signature with experimental verification in lung adenocarcinoma

**DOI:** 10.3389/fphar.2026.1936586

**Published:** 2026-07-28

**Authors:** Fan Zhang, Junyan Wang, Yu Xu, Minting Ma, Suju Wei, Chengyuan Liu

**Affiliations:** 1 Medical Oncology Department, The Fourth Hospital of Hebei Medical University, Shijiazhuang, Hebei, China; 2 General Practice Department, The Fourth Hospital of Hebei Medical University, Shijiazhuang, Hebei, China

**Keywords:** hypoxia, lung adenocarcinoma, prognostic model, single-cell RNA sequencing, tumor microenvironment, WGCNA

An incorrect **Funding** statement was provided. The correct funding statement reads:

The author(s) declared that financial support was received for this work and/or its publication. This work was supported by the Scientific Research Fund Project of Hebei Provincial Health Commission (Project Category: Youth Science and Technology Project; Tertiary Discipline: Oncological Diagnostics, Approval No. 20190763).

The original version of this article has been updated.

