# Supplementary figures and images for: Single-cell dissection and multi-cohort validation identify a hypoxia-related prognostic signature with experimental verification in lung adenocarcinoma
Source: Front Pharmacol. 2026 Jun 24;17:1845179. doi: 10.3389/fphar.2026.1845179 (PMC13342169; doi:10.3389/fphar.2026.1845179)

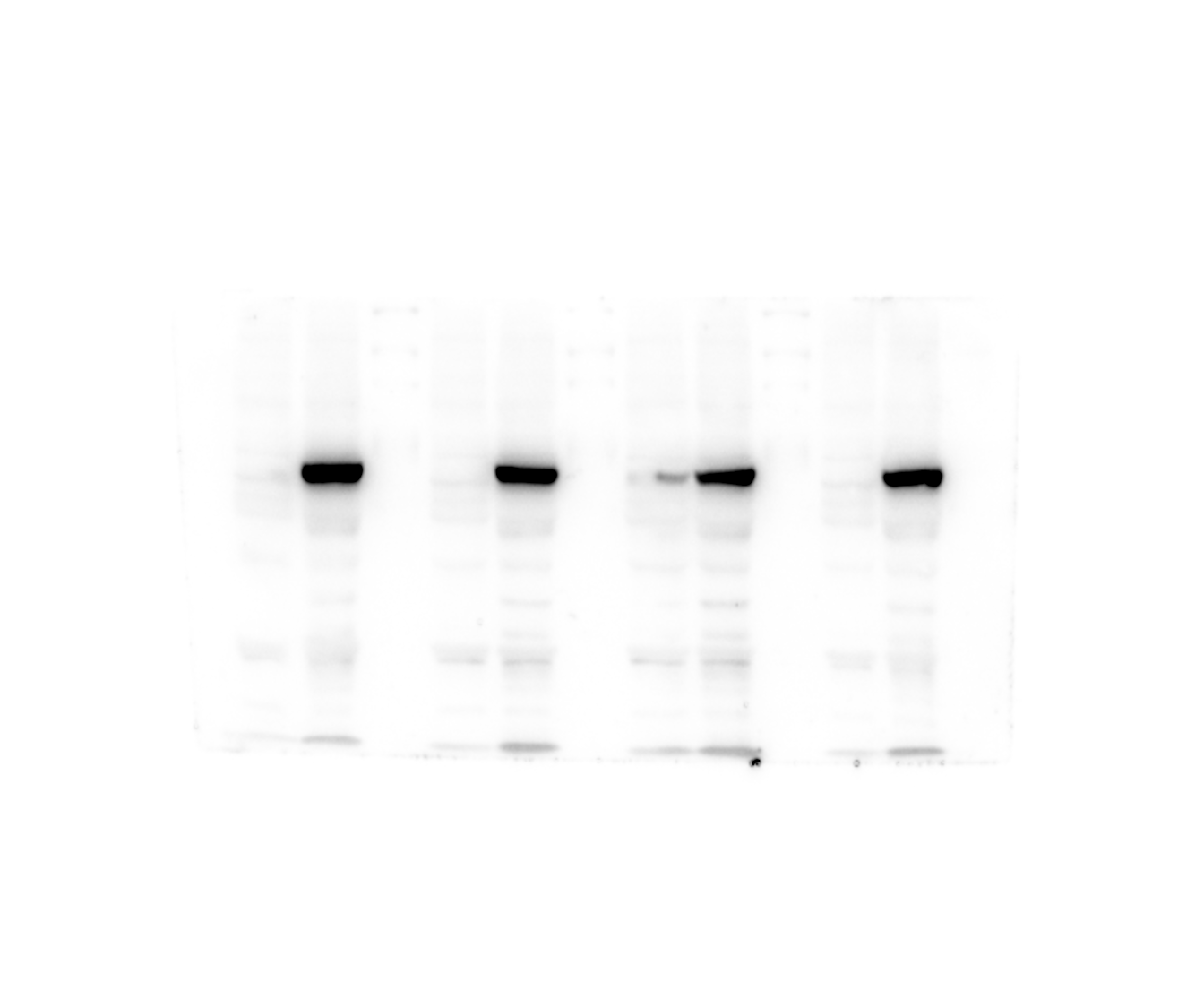

Supplement: Supplementary file 1 [file Image3.TIF]

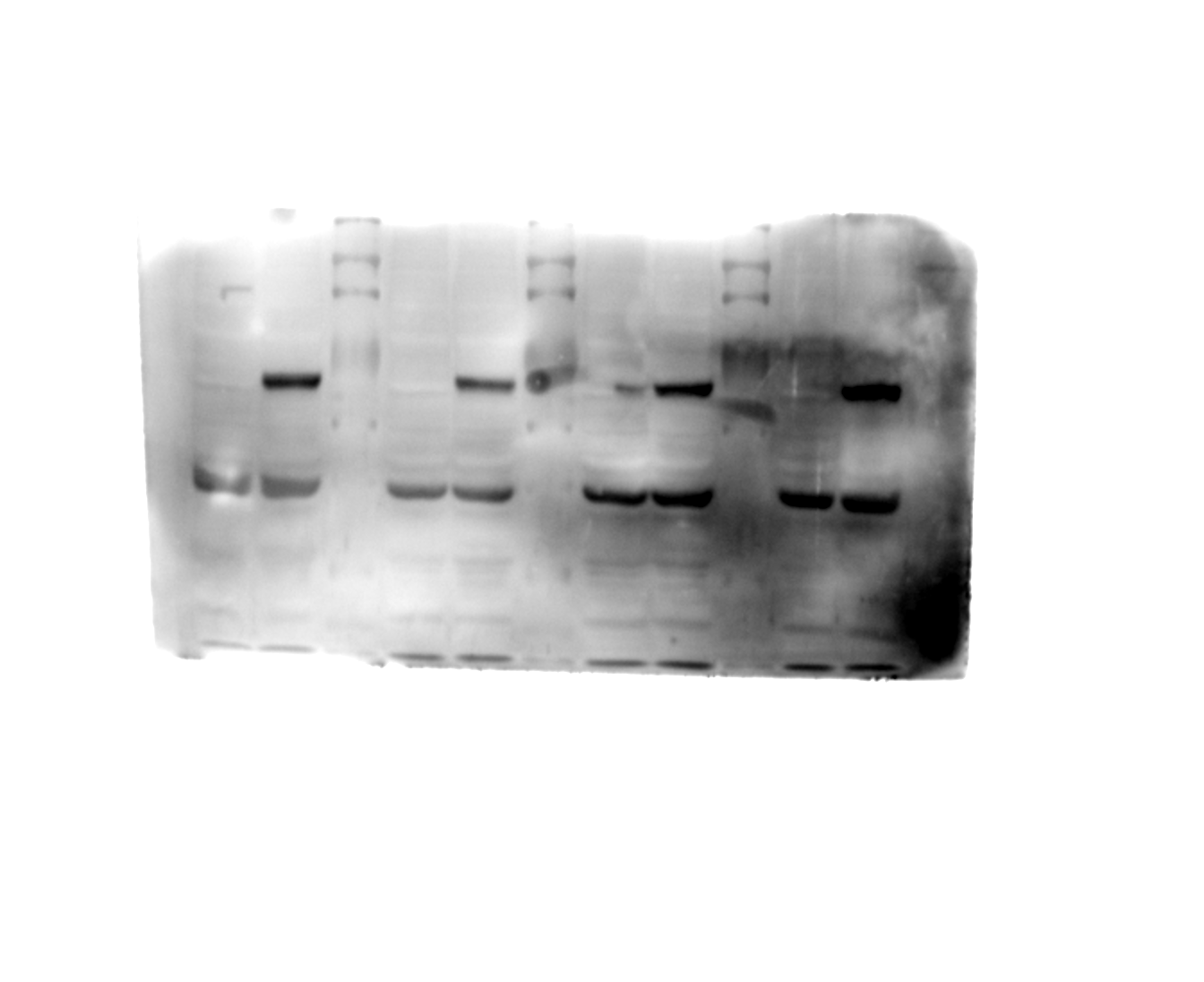

Supplement: Supplementary file 2 [file Image4.TIF]

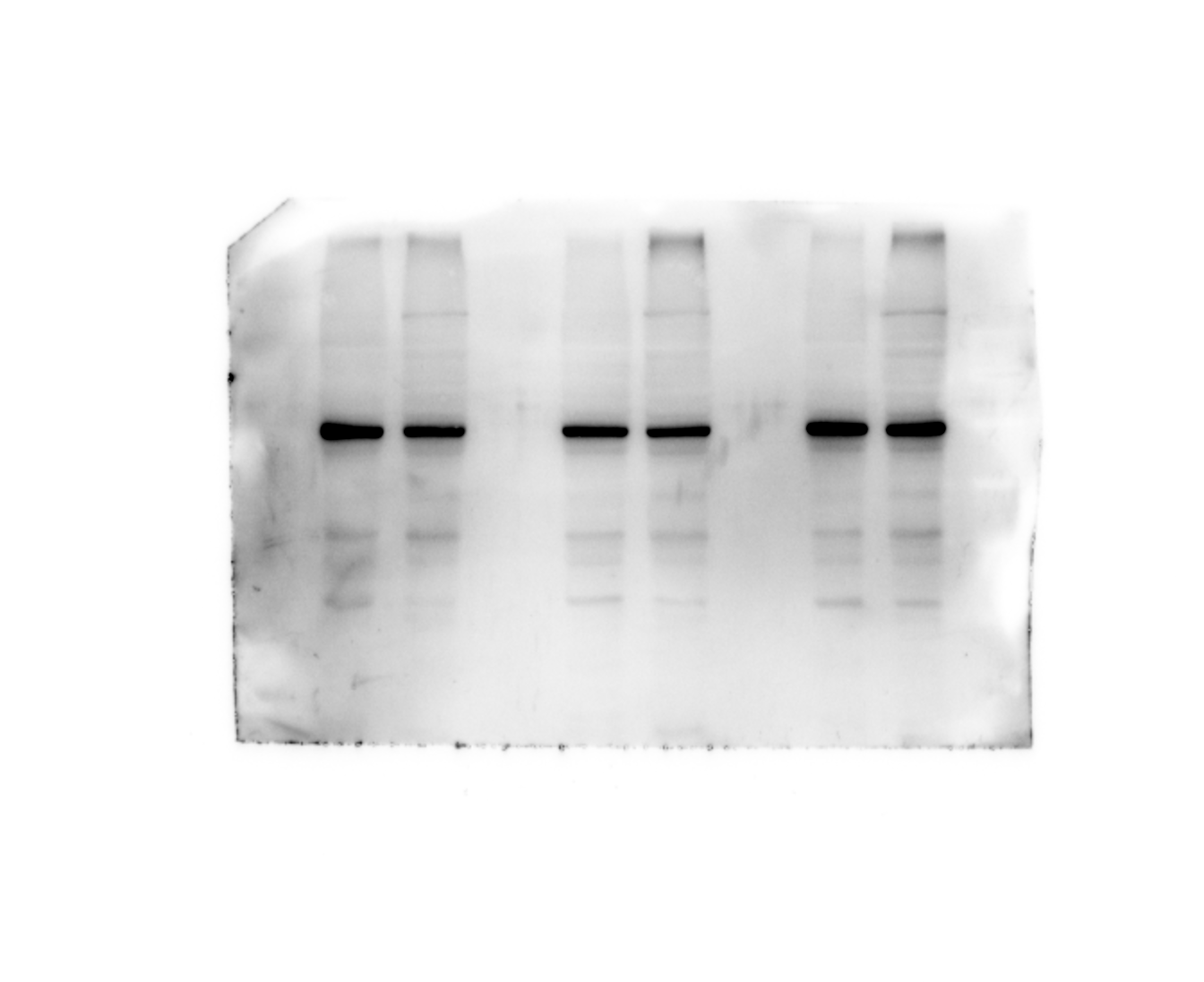

Supplement: Supplementary file 3 [file Image2.TIF]

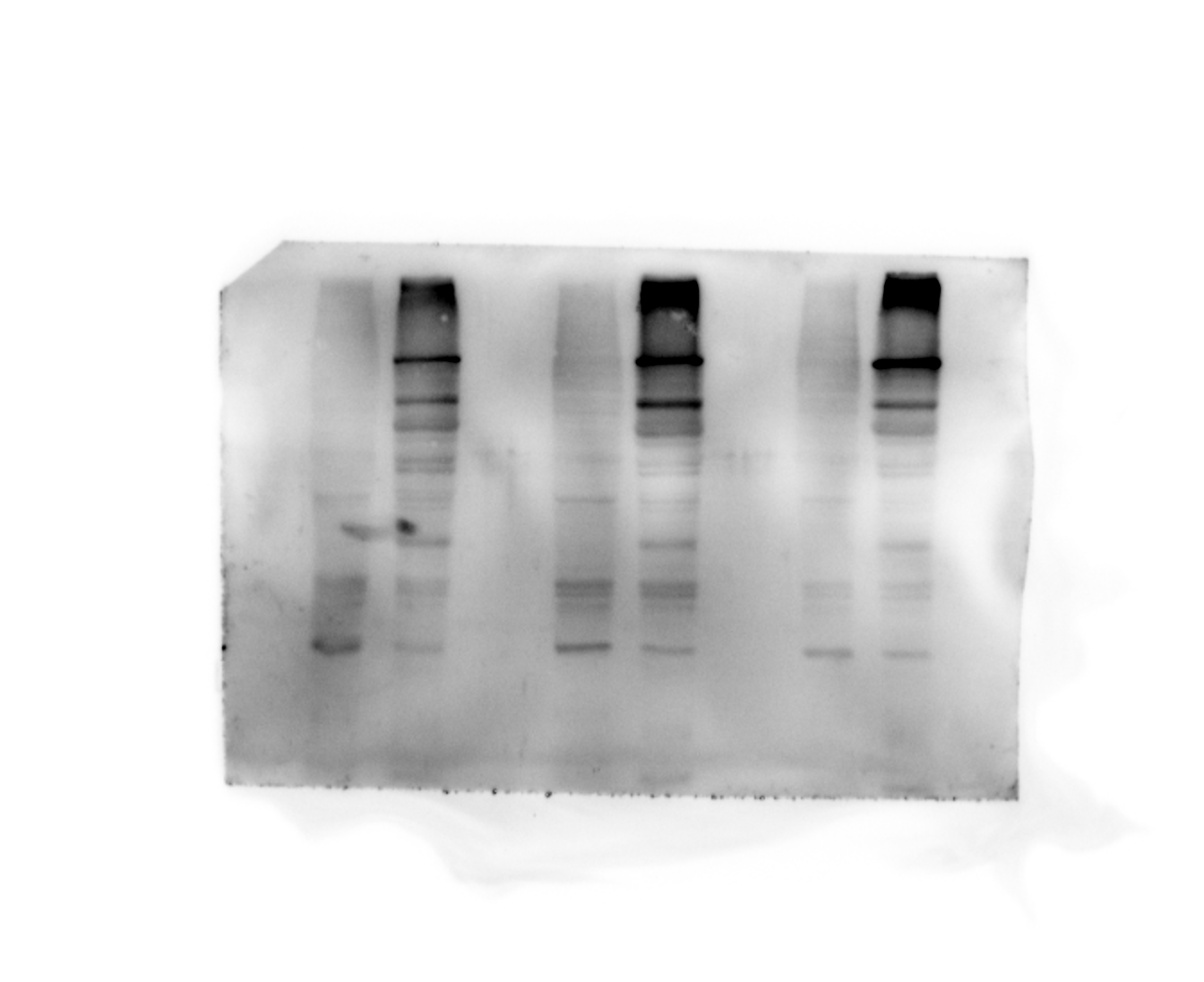

Supplement: Supplementary file 4 [file Image1.TIF]
